# Supplementary material for: A Likelihood Approach for Real-Time Calibration of Stochastic Compartmental Epidemic Models
Source: PLoS Comput Biol. 2017 Jan 17;13(1):e1005257. doi: 10.1371/journal.pcbi.1005257 (PMC5240920; doi:10.1371/journal.pcbi.1005257)
Supplement: S1 File — (TAR.GZ) [file pcbi.1005257.s014.tar.gz › HSPH_Online-SI-Revision/Additions/MSS11/n100-extreme-SEITR_noisy/n100-extreme-SEITR_noisy_NEW_table-8.pdf]

1	R0	{1.38654, 9.85565, 0.1}	{1.8801, 13.2249, 0.}	{{1.24139, 8.71343, 0.}, {2.37462, 19.3966, 0.}}	{39.3058, 42.8391, 100.}	inf	521.	303.143	{3.89286, 591.666}	56.4129	0.600591	newly	644.29	213.91	{3., 378.}	67.5698	0.831593	Reff	1.36736	1.86745	{1.24124, 2.36484}	39.9988	ar	92 379.	68 474.4	{28 211., 87 371.}	25.9028
2	R0	{2.36629, 12.2029, 0.1}	{2.01612, 12.3659, 0.}	{{1.16434, 6.25659, 0.}, {2.80387, 18.3339, 0.}}	{23.6563, 28.6181, 100.}	inf	14.	10.8423	{1.09789, 22.0387}	78.5526	0.536498	newly	20.	22.35	{0., 44.}	144.25	0.919244	Reff	2.3656	2.01568	{1.16431, 2.80236}	23.6535	ar	99 022.	63 592.2	{5., 92 604.}	35.7797
3	R0	{2.32253, 12.836, 1.}	{1.99701, 10.9532, 0.01}	{{1.18979, 3.83841, 0.}, {2.90743, 18.3339, 0.}}	{25.1023, 36.86, 99.}	inf	27.	7.34375	{0.489451, 7.01829}	94.8936	1.07587	newly	26.	116.52	{0., 28.}	503.308	1.34268	Reff	2.32139	1.99674	{1.18976, 2.90707}	25.0929	ar	98 997.	52 275.8	{1., 93 404.}	47.1946
4	R0	{1.79075, 11.2058, 1.}	{1.98659, 12.5544, 0.01}	{{1.18158, 5.58477, 0.}, {2.7499, 18.834, 0.}}	{28.834, 34.0751, 99.}	inf	97.	12.5775	{1.16209, 23.1677}	88.9925	1.26345	newly	88.	23.62	{0., 38.}	96.0682	1.44649	Reff	1.78676	1.98606	{1.18153, 2.74919}	28.9258	ar	96 920.	60 532.9	{6., 91 975.}	37.5434
5	R0	{2.26952, 9.62371, 0.1}	{2.05349, 11.2397, 0.03}	{{1.22544, 4.02511, 0.}, {2.88523, 18.834, 0.}}	{23.4891, 51.72, 124.}	inf	5.	6.24538	{0.731253, 8.02265}	105.117	0.426483	newly	5.	96.95	{0., 26.}	1929.4	0.783049	Reff	2.26934	2.05327	{1.22541, 2.88517}	23.4888	ar	98 881.	61 510.6	{2., 93 193.}	37.7933
6	R0	{1.57191, 6.89004, 0.1}	{1.96477, 11.5467, 0.02}	{{1.14639, 4.06661, 0.}, {2.93488, 18.834, 0.}}	{40.1689, 84.943, 116.}	inf	12.	15.6328	{0.784359, 14.0497}	147.522	0.632874	newly	22.	100.32	{0., 22.}	496.455	1.05295	Reff	1.57147	1.96413	{1.14635, 2.93446}	40.1498	ar	95 046.	56 834.7	{4., 93 503.}	40.2029
7	R0	{1.99226, 17.2339, 1.}	{2.17417, 13.9409, 0.}	{{1.25474, 6.00596, 0.}, {2.93978, 18.8857, 0.}}	{24.4687, 23.9184, 100.}	inf	1041.	563.763	{24.3201, 1403.12}	56.9964	0.582121	newly	648.	378.94	{27., 765.}	49.9444	0.537882	Reff	1.94486	2.1498	{1.24222, 2.8877}	24.8909	ar	97 992.	76 408.4	{37 328., 93 530.}	22.0259
8	R0	{2.63369, 15.1879, 0.1}	{2.01842, 12.8305, 0.01}	{{1.23086, 5.83416, 0.}, {2.73108, 18.9086, 0.}}	{25.7236, 27.167, 108.}	inf	52.	10.896	{1.21196, 23.7754}	82.0454	0.977636	newly	41.	16.68	{1., 44.}	92.9268	1.07112	Reff	2.6309	2.01801	{1.23082, 2.73086}	25.6791	ar	99 451.	65 857.3	{10., 91 809.}	33.7791
9	R0	{2.53271, 9.90552, 0.1}	{1.99206, 10.8677, 0.}	{{1.14344, 4.40915, 0.}, {2.91001, 18.2127, 0.}}	{27.0958, 41.0506, 100.}	inf	2.	4.81949	{0.784563, 8.39164}	171.617	0.348977	newly	8.	38.16	{0., 24.}	498.	0.894307	Reff	2.53258	1.9919	{1.14341, 2.90985}	27.0956	ar	99 355.	56 144.4	{2., 93 403.}	43.4911
10	R0	{2.48791, 18.9003, 2.}	{1.78442, 12.5312, 0.02}	{{1.12882, 5.58477, 0.}, {2.56757, 18.9003, 0.}}	{30.2738, 34.2429, 99.}	inf	311.	35.3677	{1.07657, 85.193}	90.3429	1.5115	newly	173.	45.8	{0., 140.}	88.7052	1.49373	Reff	2.47216	1.78293	{1.12867, 2.56618}	29.9766	ar	99 291.	55 456.6	{10., 90 048.}	44.1474
11	R0	{2.84414, 18.4765, 1.}	{1.76839, 13.4259, 0.}	{{1.10202, 6.0871, 0.}, {2.48143, 18.3339, 0.}}	{37.8636, 28.0249, 100.}	inf	103.	25.7297	{1.23207, 68.2719}	80.2379	1.0515	newly	76.	24.22	{1., 42.}	88.8421	1.14188	Reff	2.83774	1.76728	{1.1018, 2.47926}	37.7604	ar	99 654.	54 450.6	{9., 88 783.}	45.3603
12	R0	{2.29369, 14.622, 0.1}	{1.78841, 13.1668, 0.01}	{{1.14344, 8.14942, 0.}, {2.46794, 19.3966, 0.}}	{26.1675, 26.7265, 108.}	inf	141.	23.5711	{1.19973, 52.889}	88.2795	1.25302	newly	114.	28.26	{0., 50.}	92.7895	1.36699	Reff	2.28683	1.78745	{1.14334, 2.46607}	26.0607	ar	98 982.	57 511.6	{6., 88 860.}	41.8969
13	R0	{2.73413, 16.7529, 1.}	{2.00686, 13.0262, 0.01}	{{1.18158, 5.58477, 0.}, {2.77394, 18.4042, 0.}}	{28.0164, 26.583, 99.}	inf	78.	26.9664	{1.43278, 56.7249}	88.7384	1.00017	newly	67.	43.13	{1., 69.}	113.597	1.11674	Reff	2.72932	2.00575	{1.18152, 2.75398}	27.9368	ar	99 543.	63 793.2	{7., 92 362.}	35.914
14	R0	{1.79554, 16.5943, 2.}	{2.03546, 13.9738, 0.}	{{1.39882, 9.75208, 0.}, {2.59755, 19.1478, 0.}}	{21.6177, 21.9583, 100.}	inf	954.	460.775	{55.6916, 871.713}	55.3064	0.552061	newly	541.	306.58	{37., 617.}	49.9815	0.49096	Reff	1.75512	2.01639	{1.39702, 2.56062}	22.5324	ar	96 821.	75 215.2	{50 879., 90 383.}	22.3152
15	R0	{1.91429, 13.7771, 1.}	{1.63466, 13.7912, 0.02}	{{1.10202, 6.0871, 0.}, {2.24242, 18.9086, 0.}}	{24.061, 24.6316, 98.}	inf	379.	75.9799	{1.40809, 192.462}	81.1352	1.28681	newly	278.	47.6	{0., 147.}	83.036	1.4255	Reff	1.89691	1.63147	{1.1016, 2.23498}	23.8795	ar	97 541.	50 671.6	{14., 85 138.}	48.051
16	R0	{1.42094, 10.5871, 2.}	{1.88802, 13.8113, 0.01}	{{1.16434, 6.71734, 0.}, {2.81891, 19.5412, 0.}}	{42.526, 46.431, 99.5}	inf	380.	75.8441	{2.01825, 238.835}	82.0462	1.22475	newly	379.	67.52	{1., 193.}	85.752	1.42807	Reff	1.40736	1.88475	{1.16425, 2.8129}	43.0045	ar	93 044.	61 018.4	{16., 92 549.}	34.5159
17	R0	{1.45515, 6.16466, 0.1}	{1.9444, 10.9099, 0.}	{{1.13227, 4.00571, 0.}, {2.7499, 17.2871, 0.}}	{43.2211, 93.7584, 100.}	inf	3.	6.80124	{0.678438, 8.39103}	182.991	0.379459	newly	11.	87.39	{0., 19.}	817.545	0.958672	Reff	1.45502	1.94414	{1.13223, 2.74983}	43.2138	ar	93 254.	51 485.7	{2., 92 195.}	44.843
18	R0	{2.85669, 13.2416, 0.1}	{2.02212, 11.6879, 0.01}	{{1.23086, 5.30671, 0.}, {2.70795, 18.4504, 0.}}	{29.6539, 30.8381, 108.}	inf	11.	7.20472	{1.02636, 15.5258}	78.2036	0.574115	newly	8.	17.07	{0., 30.}	223.375	0.775847	Reff	2.85618	2.02185	{1.23085, 2.70753}	29.6505	ar	99 644.	58 480.9	{4., 91 688.}	41.3102
19	R0	{2.56417, 17.9558, 0.1}	{1.75054, 13.8042, 0.}	{{1.16434, 8.35044, 0.}, {2.48143, 19.3966, 0.}}	{32.3747, 25.6596, 100.}	inf	155.	38.4734	{1.7023, 92.824}	81.7799	1.06699	newly	91.	28.	{0., 68.}	82.5714	1.11891	Reff	2.55604	1.74894	{1.16421, 2.47729}	32.2407	ar	99 389.	55 745.2	{9., 89 021.}	43.9121
20	R0	{2.27517, 11.1864, 0.1}	{1.98939, 12.0405, 0.03}	{{1.18979, 5.83416, 0.}, {2.91001, 18.9003, 0.}}	{24.6749, 36.1754, 124.}	inf	16.	8.30991	{1.01508, 13.4148}	83.0361	0.684482	newly	17.	24.11	{0., 26.}	172.176	0.936739	Reff	2.27446	1.98906	{1.18976, 2.90922}	24.6648	ar	98 894.	54 281.5	{4., 93 121.}	45.1114
21	R0	{2.35735, 10.4735, 0.1}	{1.85755, 11.8861, 0.}	{{1.13227, 4.66877, 0.}, {2.77593, 18.4042, 0.}}	{27.8187, 43.3208, 100.}	inf	5.	5.69535	{0.321074, 8.23199}	115.16	0.528656	newly	5.	87.72	{0., 19.}	1770.	0.902779	Reff	2.35707	1.85736	{1.13223, 2.7739}	27.8143	ar	99 036.	46 625.7	{1., 90 289.}	52.9204
22	R0	{1.79551, 11.9179, 2.}	{1.83716, 12.7656, 0.}	{{1.18979, 4.02511, 0.}, {2.66225, 18.834, 0.}}	{27.2622, 35.1438, 100.}	inf	223.	29.067	{1.01232, 68.8912}	89.1587	1.39724	newly	205.	37.43	{0., 92.}	89.8878	1.54067	Reff	1.78616	1.83587	{1.18974, 2.66146}	27.3496	ar	97 033.	59 205.9	{8., 91 367.}	38.9837
23	R0	{1.23545, 6.31687, 0.1}	{1.87869, 12.8227, 0.01}	{{1.12882, 5.58477, 0.}, {2.7499, 19.1478, 0.}}	{55.3953, 111.746, 108.}	inf	123.	26.0049	{1.36569, 63.0785}	87.4126	1.21867	newly	213.	36.8	{1., 106.}	89.7277	1.58713	Reff	1.23161	1.8776	{1.12874, 2.74902}	55.67	ar	88 739.	55 402.4	{5., 91 971.}	39.075
24	R0	{2.24089, 10.1789, 0.1}	{1.83348, 11.3852, 0.}	{{1.06738, 4.06661, 0.}, {2.81891, 18.2812, 0.}}	{28.3238, 44.9791, 100.}	inf	6.	5.28928	{0.767394, 10.331}	65.3536	0.400186	newly	6.	9.28	{0., 23.}	150.	0.694601	Reff	2.24062	1.83329	{1.06718, 2.81869}	28.3233	ar	98 868.	48 208.	{3., 91 214.}	51.24
25	R0	{1.57298, 10.2607, 0.1}	{1.80775, 13.1439, 0.01}	{{1.10202, 6.00596, 0.}, {2.52368, 18.834, 0.}}	{32.0693, 42.0645, 108.}	inf	142.	30.0586	{1.1386, 82.6274}	81.0264	1.11788	newly	146.	24.25	{0., 52.}	84.9521	1.34666	Reff	1.56721	1.8065	{1.10184, 2.51744}	32.1928	ar	94 957.	56 071.8	{8., 89 640.}	40.9503
26	R0	{1.2154, 8.2576, 0.1}	{2.20065, 12.5404, 0.}	{{1.22544, 8.26611, 0.}, {2.81891, 18.2812, 0.}}	{81.9155, 59.2586, 100.}	inf	466.	404.957	{5.59546, 887.548}	59.4349	0.540469	newly	559.	348.17	{8., 800.}	57.4758	0.635235	Reff	1.19834	2.18369	{1.22169, 2.80772}	82.8937	ar	88 421.	76 616.1	{33 970., 92 818.}	15.588
27	R0	{2.22841, 18.7983, 2.}	{1.96256, 13.1181, 0.}	{{1.13726, 6.0871, 0.}, {2.81891, 18.4504, 0.}}	{24.8913, 31.0012, 100.}	inf	848.	162.726	{1.68982, 398.586}	81.5802	1.26305	newly	487.	129.27	{1., 358.}	79.1807	1.17693	Reff	2.1875	1.95557	{1.137, 2.80897}	24.7317	ar	98 756.	64 986.4	{25., 92 954.}	34.1949
28	R0	{1.27778, 5.36767, 1.}	{1.8957, 10.8967, 0.}	{{1.14639, 2.27411, 0.}, {2.7499, 17.1519, 0.}}	{52.5231, 120.81, 100.}	inf	18.	6.17856	{0.698764, 11.7594}	86.2689	0.863961	newly	37.	89.92	{0., 100.}	298.757	1.36907	Reff	1.27717	1.89549	{1.14639, 2.7498}	52.559	ar	89 843.	48 455.9	{2., 91 931.}	46.8995
29	R0	{2.38042, 15.8901, 2.}	{2.00575, 12.9932, 0.02}	{{1.13754, 5.83416, 0.}, {2.7499, 18.9086, 0.}}	{23.5388, 26.6253, 99.}	inf	126.	28.8156	{1.48174, 79.8173}	84.9657	1.14824	newly	93.	42.44	{1., 112.}	98.	1.2403	Reff	2.37421	2.00454	{1.13747, 2.74849}	23.475	ar	99 112.	64 509.9	{7., 92 200.}	34.9121
30	R0	{2.53188, 12.7607, 0.1}	{2.05888, 11.3529, 0.}	{{1.14639, 4.06661, 0.}, {2.91001, 18.8857, 0.}}	{25.2838, 35.5566, 100.}	inf	5.	7.12073	{0.608013, 8.72794}	131.102	0.478598	newly	3.	147.07	{0., 23.}	4888.33	0.779769	Reff	2.5317	2.05863	{1.14638, 2.90976}	25.2871	ar	99 317.	56 373.1	{2., 93 408.}	43.2392
31	R0	{1.24482, 10.5471, 1.}	{2.4718, 14.5112, 0.}	{{1.93539, 9.63533, 0.}, {2.96978, 18.8857, 0.}}	{98.6294, 50.9538, 100.}	inf	1273.	2164.18	{1388.12, 2921.76}	81.4825	0.267094	newly	1087.	1440.49	{1095., 1695.}	35.057	0.12804	Reff	1.19689	2.37603	{1.86729, 2.83972}	98.5158	ar	89 097.	84 208.4	{77 479., 93 874.}	11.1372
32	R0	{2.30446, 17.9607, 1.}	{1.81227, 13.3288, 0.}	{{1.14639, 6.0871, 0.}, {2.70795, 18.834, 0.}}	{27.7234, 27.9587, 100.}	inf	427.	30.4221	{1.3882, 76.9418}	92.8754	1.6722	newly	254.	38.15	{0., 70.}	91.0118	1.70103	Reff	2.28427	1.81101	{1.14634, 2.6961}	27.4482	ar	99 028.	57 740.9	{7., 91 721.}	41.6924
33	R0	{2.31579, 11.8236, 2.}	{2.04032, 11.8491, 0.01}	{{1.28596, 4.06661, 0.}, {2.77394, 18.4042, 0.}}	{21.4005, 35.7232, 99.5}	inf	28.	3.74459	{0.85895, 5.92062}	89.2014	1.11607	newly	33.	16.24	{0., 13.}	120.909	1.4405	Reff	2.31442	2.0402	{1.28585, 2.77354}	21.3912	ar	99 012.	48 450.9	{1., 91 845.}	51.0657
34	R0	{1.6678, 10.6744, 1.}	{1.83084, 13.0563, 0.02}	{{1.16434, 6.28417, 0.}, {2.53075, 18.4504, 0.}}	{26.0857, 35.9779, 98.}	inf	242.	33.2587	{1.15348, 95.3717}	87.4674	1.40502	newly	267.	42.22	{0., 125.}	90.1348	1.66071	Reff	1.65861	1.82945	{1.16426, 2.52671}	26.2803	ar	95 809.	58 389.5	{7., 89 736.}	39.0564
35	R0	{1.65476, 7.07559, 0.1}	{2.06933, 11.6001, 0.}	{{1.22252, 4.02511, 0.}, {2.96265, 18.8857, 0.}}	{36.6094, 83.155, 100.}	inf	8.	12.3209	{0.65884, 8.82372}	168.354	0.603458	newly	10.	403.5	{0., 35.}	4058.4	1.01553	Reff	1.65458	2.06884	{1.2225, 2.95979}	36.5902	ar	95 746.	55 503.5	{2., 93 397.}	42.0305
36	R0	{2.71807, 18.5959, 0.1}	{1.87585, 13.2596, 0.}	{{1.13726, 6.0871, 0.}, {2.73108, 19.3966, 0.}}	{32.4314, 30.2704, 100.}	inf	89.	20.8678	{1.83121, 49.0464}	79.4351	0.978496	newly	61.	17.54	{0., 37.}	81.7377	1.05839	Reff	2.71258	1.87499	{1.13709, 2.72901}	32.3545	ar	99 540.	56 115.	{11., 91 906.}	43.6256
37	R0	{2.6202, 12.7552, 0.1}	{1.88031, 12.8158, 0.01}	{{1.14639, 6.00596, 0.}, {2.6365, 18.4042, 0.}}	{30.0347, 28.7478, 108.}	inf	7.	18.5761	{1.34194, 27.639}	220.535	0.459131	newly	7.	34.85	{0., 30.}	477.286	0.678008	Reff	2.61991	1.87955	{1.14635, 2.63534}	30.0442	ar	99 426.	56 154.7	{6., 90 858.}	43.5211
38	R0	{1.47672, 9.81651, 1.}	{1.88532, 13.4002, 0.01}	{{1.18158, 8.35044, 0.}, {2.73108, 18.2127, 0.}}	{37.8523, 45.9059, 99.}	inf	332.	88.3595	{1.51318, 223.581}	79.3596	1.13271	newly	329.	81.98	{1., 217.}	84.3526	1.36645	Reff	1.46491	1.8816	{1.18147, 2.72163}	38.1986	ar	93 815.	63 215.1	{18., 91 892.}	32.6225
39	R0	{1.90004, 12.3313, 1.}	{1.8783, 13.2766, 0.02}	{{1.14344, 5.71232, 0.}, {2.6365, 18.9086, 0.}}	{23.6414, 30.7081, 98.}	inf	172.	21.7195	{2.09105, 50.8065}	87.3724	1.22027	newly	167.	23.79	{1., 53.}	88.7246	1.41693	Reff	1.89271	1.87741	{1.14334, 2.63491}	23.7156	ar	97 413.	65 138.7	{15 498., 90 929.}	33.1314
40	R0	{2.64218, 18.5237, 2.}	{1.85994, 12.1128, 0.}	{{1.14344, 4.06661, 0.}, {2.70795, 17.1868, 0.}}	{31.5606, 35.392, 100.}	inf	279.	26.9454	{1.2264, 62.1307}	91.0346	1.53183	newly	187.	39.31	{0., 114.}	90.0053	1.55637	Reff	2.62693	1.85882	{1.14339, 2.70449}	31.3345	ar	99 474.	55 831.5	{8., 91 826.}	43.8732
41	R0	{1.6945, 7.17996, 0.1}	{2.05332, 11.5597, 0.01}	{{1.22544, 4.06661, 0.}, {2.86001, 18.9086, 0.}}	{32.6322, 81.0281, 108.}	inf	5.	8.38313	{0.572366, 8.30665}	159.158	0.514034	newly	9.	269.29	{0., 24.}	3013.22	0.98749	Reff	1.69433	2.053	{1.22525, 2.85978}	32.6229	ar	96 332.	57 066.1	{1., 92 758.}	40.761
42	R0	{1.84559, 18.0074, 1.}	{2.47384, 14.6894, 0.}	{{1.28596, 3.98513, 0.}, {2.96978, 18.8857, 0.}}	{40.4685, 21.4898, 100.}	inf	1558.	1080.68	{204.816, 1657.93}	33.923	0.256534	newly	870.	709.81	{363., 985.}	25.6057	0.151197	Reff	1.78061	2.4264	{1.27833, 2.90411}	42.2627	ar	97 358.	84 068.3	{42 741., 93 860.}	13.6504
43	R0	{2.24965, 17.0361, 2.}	{1.87492, 13.0662, 0.}	{{1.14344, 8.26611, 0.}, {2.53075, 18.9003, 0.}}	{23.2756, 27.6358, 100.}	inf	242.	34.0398	{1.40725, 65.3946}	90.0797	1.37805	newly	161.	38.04	{1., 84.}	91.677	1.40029	Reff	2.23721	1.87347	{1.14336, 2.5275}	23.1282	ar	98 770.	60 424.9	{18., 89 850.}	38.8226
44	R0	{2.54324, 15.7967, 0.1}	{1.83262, 13.2646, 0.02}	{{1.10202, 6.0871, 0.}, {2.59755, 18.9003, 0.}}	{30.6656, 25.1355, 116.}	inf	117.	26.9491	{1.35113, 61.7344}	86.4068	1.13239	newly	107.	31.07	{0., 54.}	96.215	1.34441	Reff	2.53714	1.83149	{1.10185, 2.5967}	30.5625	ar	99 354.	56 275.3	{9., 90 517.}	43.3588
45	R0	{2.54943, 14.8032, 0.1}	{2.0118, 13.0636, 0.01}	{{1.14639, 6.48739, 0.}, {2.9194, 18.4042, 0.}}	{26.364, 26.4419, 108.}	inf	57.	11.3169	{1.21836, 28.0643}	80.8032	0.997982	newly	50.	15.27	{0., 36.}	83.9	1.18721	Reff	2.54637	2.01135	{1.14638, 2.91777}	26.3305	ar	99 380.	60 855.3	{4., 93 527.}	38.765
46	R0	{2.37755, 12.6461, 2.}	{2.01186, 10.8386, 0.01}	{{1.23086, 4.00571, 0.}, {2.74056, 18.4042, 0.}}	{21.68, 35.0437, 99.5}	inf	16.	4.39803	{0.938353, 8.31682}	79.3045	0.809938	newly	26.	29.42	{0., 40.}	164.769	1.18728	Reff	2.37696	2.01172	{1.23085, 2.74042}	21.6731	ar	99 096.	52 664.3	{1., 91 402.}	46.8553
47	R0	{1.69279, 11.6636, 1.}	{1.85, 13.0243, 0.}	{{1.13754, 8.26611, 0.}, {2.6365, 18.8857, 0.}}	{27.5826, 30.9261, 100.}	inf	202.	34.1802	{2.05829, 76.6268}	84.803	1.14935	newly	167.	28.2	{1., 66.}	85.2335	1.24446	Reff	1.68417	1.84857	{1.13745, 2.63297}	27.776	ar	96 214.	60 219.8	{19., 90 942.}	37.4105
48	R0	{1.82402, 10.0038, 1.}	{1.93108, 12.9965, 0.}	{{1.14639, 6.25659, 0.}, {2.7499, 18.4042, 0.}}	{26.2802, 45.0435, 100.}	inf	33.	9.89638	{1.02696, 24.0593}	79.5552	0.883694	newly	39.	15.64	{0., 34.}	97.6923	1.24972	Reff	1.82252	1.93069	{1.14636, 2.74954}	26.2981	ar	97 076.	60 057.6	{6., 92 039.}	38.1335
49	R0	{1.6424, 11.1667, 2.}	{1.92416, 11.806, 0.}	{{1.13726, 5.43578, 0.}, {2.74056, 18.4042, 0.}}	{32.5733, 36.3434, 100.}	inf	392.	74.4167	{1.87303, 247.468}	82.6509	1.31374	newly	380.	105.94	{1., 390.}	80.1421	1.40509	Reff	1.62686	1.92106	{1.1371, 2.72547}	33.0003	ar	95 546.	61 919.5	{9., 91 804.}	35.1941
50	R0	{2.05638, 14.3048, 1.}	{1.8962, 12.2258, 0.}	{{1.13754, 5.30671, 0.}, {2.66225, 18.4042, 0.}}	{23.1073, 28.4502, 100.}	inf	221.	22.7725	{1.20833, 45.0943}	91.272	1.48071	newly	176.	33.41	{0., 76.}	92.4602	1.53607	Reff	2.0461	1.89521	{1.13745, 2.66198}	23.0888	ar	98 194.	59 696.	{9., 91 206.}	39.2061
